# Preoperative patient factors associated with blood product use in cardiac surgery, a retrospective cohort study

**DOI:** 10.1186/s13019-022-01770-5

**Published:** 2022-02-23

**Authors:** Aditya Eranki, Ashley Wilson-Smith, Umar Ali, Christopher Merry

**Affiliations:** 1 Department of Cardiothoracic Surgery, John Hunter Hospital, Newcastle, Australia; 2 The Collaborative Research (CORE) Group, Macquarie University, Sydney, Australia; 3 Department of Cardiothoracic Surgery, Fiona Stanley Hospital, Perth, Australia; 4 Medical School, University of Western Australia, Perth, Australia; 5 Medical School, University of Notre Dame, Perth, Australia

**Keywords:** Cardiac surgery, Blood transfusion, Blood product use, Coronary artery bypass graft, Valvular heart surgery, Risk factors

## Abstract

**Background:**

Cardiac surgery is associated with a high rate of blood use. The aim of this study is to identify preoperative patient factors associated with allogeneic Red Blood Cell (RBC) or non-Red Blood Cell (NRBC) use in cardiac surgery.

**Methods:**

All adult cardiac surgical procedures conducted at a single Western Australian institution were retrospectively analysed. Data was collected from the Australia and New Zealand Cardiac Surgery Database from 2015 to 2018. A number of preoperative factors were identified, relating to past medical history or preoperative cardiac status. Outcome 1 was defined as the use of one or more RBC products intra or post-operatively. Outcome 2 was defined as the use of one or more NRBC products intra or post-operatively. Multivariate logistical regression analysis was done to assess for the association between preoperative factors and allogeneic blood product use.

**Results:**

A total of 1595 patients were included in this study, of which 1488 underwent a Coronary Artery Bypass Graft, Valve or a combined procedure. Patients on dialysis preoperatively and those who had preoperative cardiogenic shock demonstrated the greatest risk of requiring RBC transfusion with an odds ratio of 5.643 (95% CI 1.305–24.40) and 3.257 (95% 1.801–5.882) respectively. Patients who had preoperative cardiogenic shock demonstrated the greatest risk of requiring NRBC transfusion with an odds ratio of 3.473 (95% CI 1.970–6.135). Patients who have had a previous cardiothoracic intervention are at increased risk of both RBC and NRBC transfusion, with adjusted odds ratios of 1.774 (95% CI 1.353–2.325) and 2.370 (95% CI 1.748–3.215) respectively.

**Conclusion:**

A number of factors relating to past medical history or preoperative cardiac status are implicated with increased allogeneic blood product use in cardiac surgery. Identifying high-risk patients in a preoperative setting can enable us enrol them in a blood conservation program, therefore minimizing the risk of exposure to blood transfusion.

## Introduction

Cardiac surgery is associated with a high rate of blood product use. The rate of blood transfusion varies greatly amongst different centres, from between 20 and 67% [1]. In Australia, cardiac surgery accounts for the second most common surgical indication for packed red blood cell (RBC) use, the largest indication for fresh frozen plasma (FFP) and the second largest indication for platelet transfusion after hematological and oncological disorders [2, 3]. This represents a large economic burden as cross matching and administration of blood has both financial and resource-associated costs [4]. The transfusion of blood products in patients is also associated with adverse events following cardiac surgery [5–7]. Therefore, the use of blood products after cardiac surgery should be carefully considered. There has been extensive study in identifying the need to transfuse, by development of several scoring methods [8–12]. Most of these models have not been externally validated and are of uncertain reliability. A multitude of pre and peri operative factors have been identified and associated with blood product use in cardiac surgery, again with varying levels of significance [1, 4, 10, 12–18]. It is important to identify these factors in order to risk stratify patients undergoing cardiac surgery. The objective of this study is to identify preoperative factors associated with allogenic blood product use in cardiac surgery at a single West Australian institution.

## Methods

### Patients

Data was retrospectively collected from the Australian and New Zealand Cardiac Surgery database (ANZSCTS). All patients undergoing cardiac procedures from 2015 to 2018 from a single Western Australian institution were included. Preoperative factors were identified, hypothesized to be associated with an increased risk of transfusion. Patient related factors: age, body mass index (BMI), Diabetes Mellitus, dialysis at the time of operation, history of smoking, total body surface area (TBSA), presence of respiratory disease, and gender were assessed. Preoperative cardiac status: New York Heart Association (NYHA) class, preoperative haemoglobin (Hb), anticoagulant use within 24 h of surgery, non-valve/coronary artery bypass procedures, previous cardiac intervention, preoperative cardiogenic shock, preoperative antiplatelet use within 24 h, and preoperative creatinine clearance were also assessed. These are summarised in Table 1. The definitions of the preoperative factors are in accordance with those set by the ANZCTS database. Patients were considered to have had a transfusion if they received blood products intra-operatively or during their post-operative stay.

**Table 1:** Preoperative factors

Patient related factors	Preoperative cardiac status
Body mass index (BMI m/kg^2^)	New York Heart Association (NYHA) class
Age (years)	Preoperative haemoglobin (g/L)
Diabetes mellitus (yes/no)	Anticoagulant use less than 24 h preoperatively (yes/no)^c^
Renal dialysis at the time of operation (yes/no)	Non Valve/CABG procedure (yes/no)
History of smoking (yes/no)	Previous cardiothoracic intervention (yes/no)
Total body surface area^a^ (TBSA m^2^)	Presence of cardiogenic shock preoperatively
Presence of respiratory disease^b^ (yes/no)	Antiplatelet less than 24 h preoperatively^d^
Gender (male/female)	Preoperative creatinine clearance

^a^TBSA is calculated by 0.007184 × HTM^0.725^ × WKG^0.42^ where weight (WKG) and height (HTM) are available. Units in m^2^

^b^Either mild: patient is on chronic inhaled or oral bronchodilator therapy moderate: patient is on chronic oral steroid therapy directed at lung disease. Severe: p02 on room air < 60 or pC02 on room air > 50 or mechanical ventilation for chronic lung disease

^c^The use of warfarin/heparin/LMWH/thrombin inhibitors and or factor Xa inhibitors within 24 h of surgery

^d^The use of Ticagrelor or Clopidogrel within 24 h of surgery

### Transfusion triggers

All procedures were performed by surgeons at a single institution. Hematocrit levels of 20% during CPB and 25% after CPB into the postoperative period were accepted if the patient had no evidence of ongoing bleeding. Intraoperatively, all attempts were made to hemoconcentrate. If there was intolerable anemia, cell saver blood was returned first to the patient prior to the use of allogenic blood transfusion. Postoperatively, the triggers for transfusion were hemodynamic instability associated with evidence of ongoing bleeding. In most patients, a hemoglobin target of greater than 7 g/dl was accepted. The use of NRBC was guided by the degree of intraoperative blood loss and thromboelastography (TEG).

### Statistical methods

Two outcomes were considered. Outcome 1 was defined as the use of one or more units of RBC either intra-operatively or throughout the period of hospitalization. Outcome 2 was defined as the use of NRBC, namely fresh frozen plasma (FFP), Platelets or Cryoprecipitate. A further subgroup analysis was performed, assessing factors associated with large volume transfusion (i.e., defined as greater than four units of PRBC). Descriptive statistics, including mean, number and standard deviation were calculated for each preoperative factor. Univariate analysis was conducted to identify variables significantly associated with allogenic transfusions. Categorical variables were assessed using the chi-squared (x^2^) test to ascertain odds ratio. Cramer’s V coefficients were also calculated to assess the strength of association of categorical variables. Continuous variables were first assessed for normality and then with the independent T test to assess for equality of means. Then, univariate logistical regression was done to ascertain odds ratios for continuous variables. P values less than 0.05 were deemed as significant. Preoperative factors that reached significance in univariate analysis were then further assessed with multivariate logistical regression analysis using a binary logistic regression model. Adjusted odds ratios were obtained and P values less than 0.05 were denoted as statistically significant. All analysis was done on *SPSS statistics* version 25.

A total of 1595 patients were included in the analysis. Preoperative hemoglobin (Hb) had 629 missing data points out of the total of 1595. A separate statistical analysis was conducted to ascertain odds ratios and significance for preoperative Hb in both univariate and multivariate analysis. All other preoperative factors had the full complement of data points available.

## Results

Baseline patient data is summarised in Table 2. A total of 1595 patients were included in the retrospective analysis. Most of these patients underwent coronary artery bypass grafting (CABG), valve or combined procedures with a total of 1386. Of the 1595 patients, 447 required at least one unit of RBC either intra-operatively or during their hospital admission. A total of 298 patients required at least one unit of platelets, cryoprecipitate or FFP (NRBC).

**Table 2:** Baseline data

Patient demographics	Mean ± SD/number
Gender	Male = 1158 Female = 437
Age	62.7 ± 13.3
Preoperative creatinine clearance (g/DL)	104.8 ± 98.1
Respiratory disease	No disease = 1343 Disease present = 252
Dialysis	Dialysis = 33 No dialysis = 1562
Diabetes mellitus	No diabetes = 1051 Diabetes = 544
Hx of smoking	Non-smoker = 558 Smoker = 1037
BMI (kg/m^2^)	29.0 ± 6.53
TBSA (m^2^)	1.91 ± 0.22
NYHA	
Class 1 Class 2 Class 3 Class 4	697 487 326 85
Preoperative Hb (g/L)	131.3 ± 21.7
Antiplatelet use < 24 h	Yes = 223 No = 1372
Anticoagulant use < 24 h	Yes = 361 No = 1234
Non valve/CABG procedure	Yes 209 No = 1386
Previous cardiothoracic intervention	Yes = 280 No = 1315
Presence of cardiogenic shock preoperatively	Yes = 52 No = 1541

### RBC transfusions

Several variables were significantly associated with outcome 1 in the multivariate analysis. These are summarised in Table 3. Age, creatinine clearance, preoperative Hb, the presence of cardiogenic shock preoperatively (*P* < 0.001) and total body surface area (*P* = 0.002) were most significantly associated with RBC transfusion in Cardiac surgery.

**Table 3:** Univariate and multivariate analysis for RBC transfusion (outcome 1)

Factor	Odds ratio (95% CI)	*P* value	Adjusted odds ratio	*P* value
Gender (female vs. male)	1.678 (1.325–2.126)	*P* < 0.001	1.536 (1.082– 2.053)	*P* = 0.004
Age (per year increase)	1.017 (1.009–1.026)	*P* < 0.001	1.018 (1.009–1.027)	*P* < 0.001
Creatinine (per µmol/L increase)	1.004 (1.002–1.005)	*P* < 0.001	1.006 (1.004–1.009)	*P* < 0.001
Respiratory disease ( yes vs. no )	1.183 (0.883–1.586)	*P* = 0.259		
Dialysis (Yes vs No)	3.596 (1.787–7.236)	*P* < 0.001	5.643 (1.305–24.398)	*P* = 0.021
Diabetes ( yes vs. no )	1.094 (0.870–1.376)	*P* = 0.442		
Smoking (yes vs. no)	0.866 (0.690–1.087)	*P* = 0.214		
BMI (per kg/m^2^ increase)	0.975 (0.956–0.994)	*P* = 0.010	0.991 (0.971–1.011)	*P* = 0.366
TBSA (per m^2^ increase)	0.251 (0.150–0.419)	*P* < 0.001	0.353 (0.178–0.700)	*P* = 0.003
NYHA				
Class 1	1.115 (0.895–1.390)	*P* = 0.330		
Class 2	0.869 (0.683–1.105)	*P* = 0.251		
Class 3	0.974 (0.742–1.279)	*P* = 0.851		
Class 4	1.140 (0.709–1.833)	*P* = 0.589		
Preoperative Hb (per g/L increase)	0.972 (0.965–0.978)	*P* < 0.001	0.978 (0.970–0.985)	*P* < 0.001
Antiplatelet use < 24 h ( yes vs. no )	1.039 (0.760–1.422)	*P* = 0.809		
Anticoagulant use < 24 h ( yes vs. no )	1.476 (1.147–1.898)	*P* = 0.002	1.302 (0.999–1.701)	*P* = 0.053
Non valve/CABG (yes vs. no)	1.641 (1.210–2.225)	*P* < 0.001	1.567 (1.133–2.164)	*P* = 0.007
Previous cardiothoracic intervention (yes vs. no)	1.774 (1.353–2.325)	*P* < 0.001	1.563 (1.169–2.087)	*P* = 0.004
Presence of cardiogenic shock preoperatively (yes vs. no)	3.645 (2.101–6.324)	*P* < 0.001	3.257 (1.801–5.882)	*P* < 0.001

The average age of patients who received RBC transfusion was 64.7 years compared to 61.8 years in the non-RBC cohort. The adjusted odds ratio for every year increase in age was 1.018 (95%CI 1.009–1.027). The average preoperative creatinine clearance in RBC transfused patients was 130.3 µmol/L compared to 94.9 µmol/L in non-transfused patients, presenting an adjusted odds ratio of 1.006 (95%CI 1.004–1.009) for every unit increase in creatinine clearance. The average preoperative Hb in the transfused cohort of patients was 122.07 g/L compared to 135.39 g/L with an odds ratio of 0.978 (95%CI 0.970–0.985) for every unit increase in Hb. Of those with cardiogenic shock preoperatively, 57.4% required transfusion of RBC compared to 27% of those without cardiogenic shock. The adjusted odds ratio was 3.257 (95%CI 1.801–5.882). A low TBSA was also highly associated with RBC transfusion, with an average TBSA of 1.86 m^2^ in transfused patients versus 1.92 m^2^ in non-transfused patients. The adjusted odds ratio of requiring RBC transfusion was 2.83 (95% 1.423–5.618) for every unit m^2^ decrease in TBSA.

Other patient factors associated with RBC transfusion on multivariate analysis were female gender, haemodialysis, anticoagulant use within 24 h of operating, non-Valve/CABG procedures and history of previous cardiothoracic interventions. Female gender was associated with a 1.536 (95%CI 1.082–2.053) fold increased risk of requiring a transfusion. Non CABG/Valve procedures, encompassing aortic surgery and trauma, was positively associated with RBC transfusion, with an odds ratio of 1.641 (95%CI 1.210–2.225). A history of previous cardiothoracic intervention was also associated with an increased risk of transfusion, with an adjusted odds ratio of 1.563 (95%CI 1.169–2.087). Patients on haemodialysis were associated with the greatest risk of transfusion with an adjusted odds ratio of 5.643 (95%CI 1.305–24.398), however, out of the 1595 patients included in the analysis, only 33 were on haemodialysis preoperatively.

Body mass index reached significance in univariate analysis however failed to reach significance on subsequent multivariate analysis. The presence of respiratory disease, diabetes mellitus, history of smoking, and antiplatelet use within 24 h were not associated with increased risk of RBC transfusion in our analysis.

### Non-RBC transfusions

**Fig. 1:**
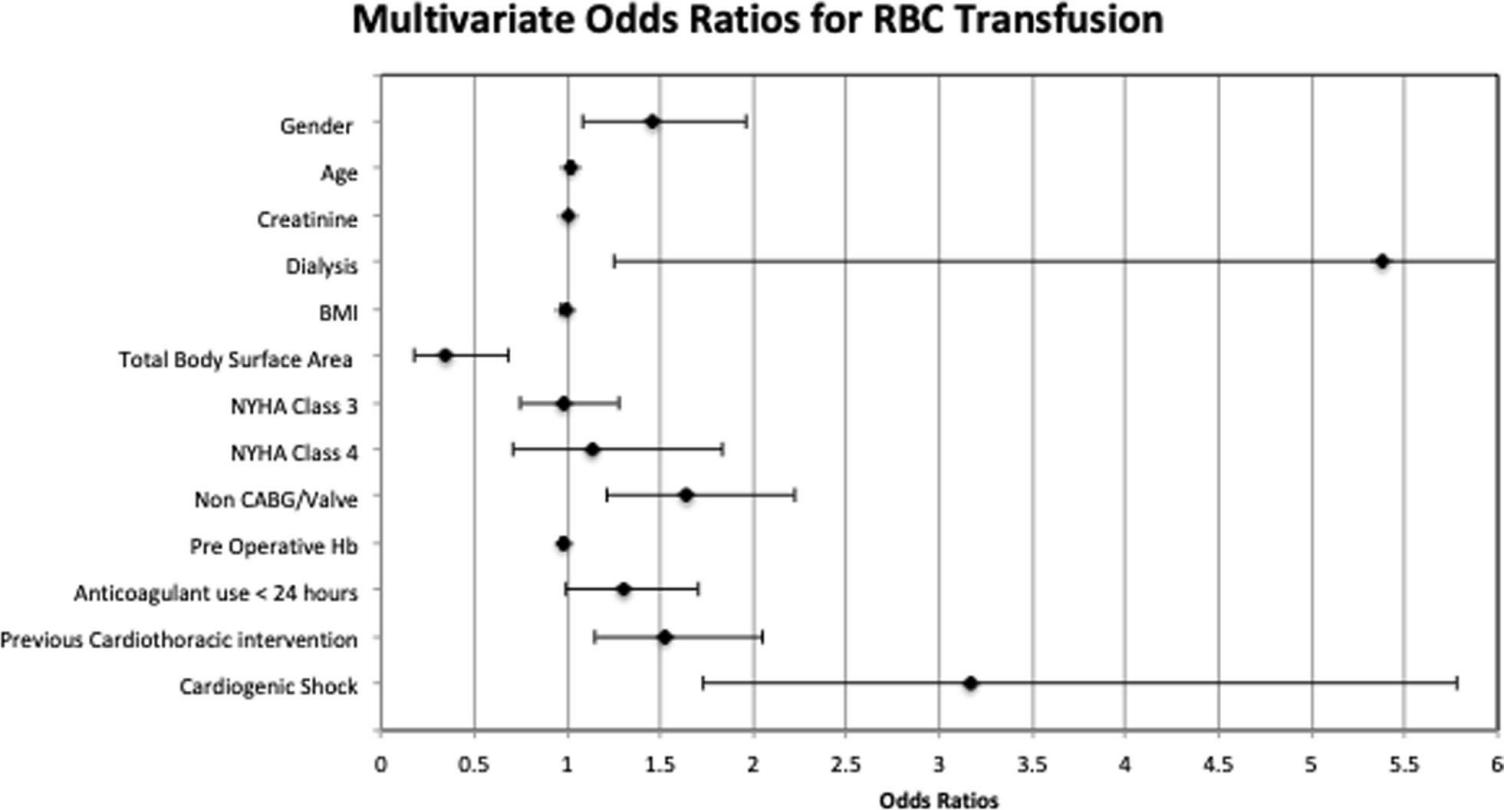
Forrest plot; multivariate odds ratios for RBC transfusion

**Fig. 2:**
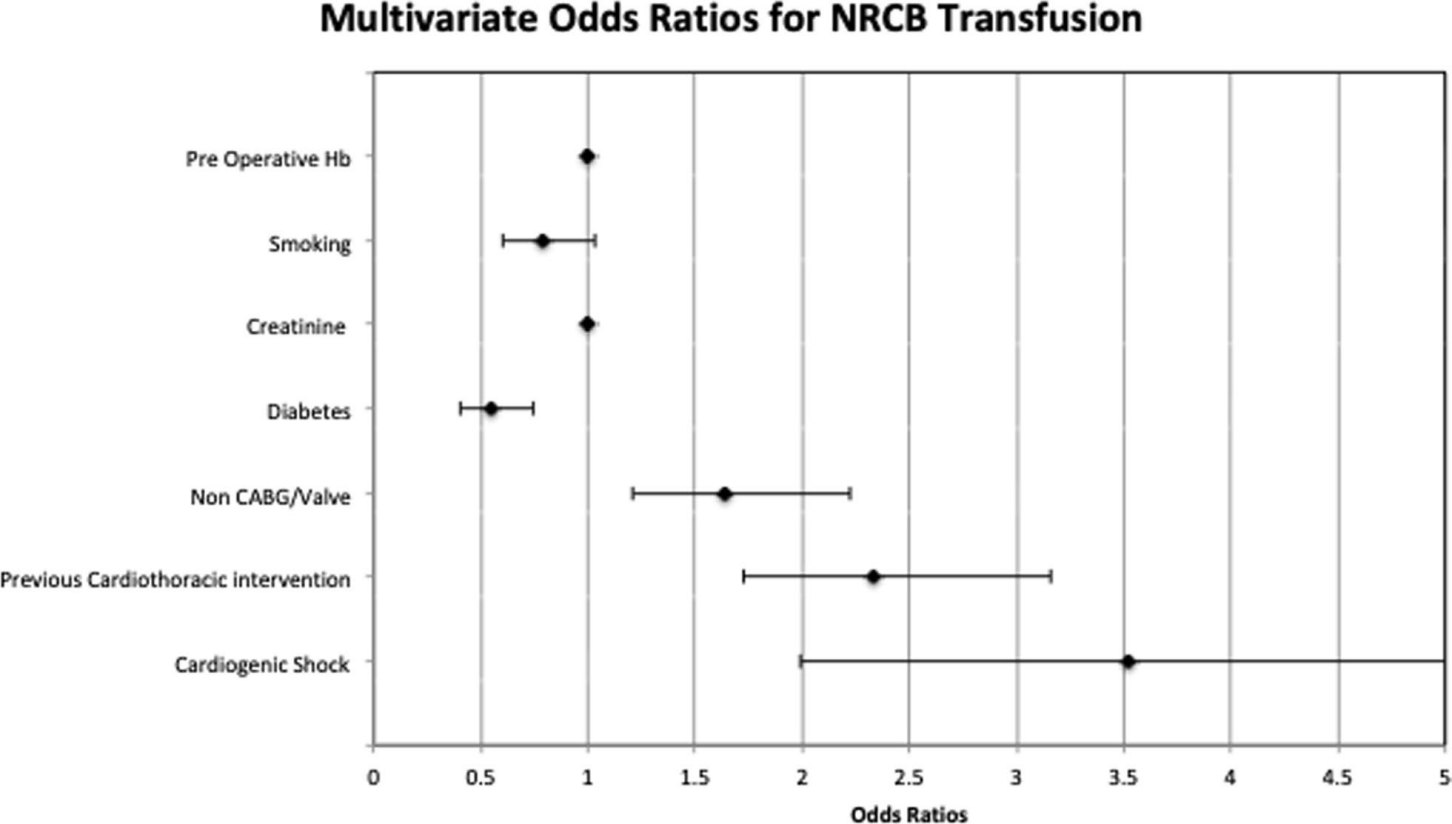
Forrest plot; multivariate odds ratios for NRBC transfusion

Factors significantly associated with NRBC transfusion are summarised in Table 4. Mean preoperative creatinine clearance was 102.1 and 116.9 g/dL in patients that did not require NRBC transfusion compared to those who did respectively. The odds ratio was 1.001 (95% CI 1.000–1.002) for every unit increase in creatinine clearance. Previous history of cardiothoracic intervention and the presence of cardiogenic shock preoperatively were both associated with NRBC transfusion, with odds ratios of 2.370 (95% CI 1.748–3.215) and 3.473 (95% CI 1.970–6.135) respectively. The presence of cardiogenic shock was the strongest predictor of NRBC transfusion in cardiac surgery, with 50% of these patients requiring NRBC products. Interestingly, the presence of diabetes mellitus in the cohort of patients undergoing cardiac surgery was significantly associated with a decreased risk of NRBC transfusion. As such, patients with diabetes mellitus preoperatively had an adjusted odds ratio of 0.536 (95% CI 0.397–0.724). Non-CABG/Valve procedures demonstrated significance on univariate analysis with an Odds ratio of 1.641 (95% 1.210–2.225) however failed to demonstrate an association with NRBC transfusion on multivariate analysis. Adjusted odds ratios have been graphically demonstrated on Forrest plots, in Figs. 1 and 2 below.

**Table 4:** Univariate and multivariate analysis for NRBC transfusion (outcome 2)

Factor	Odds ratio (95% CI)	*P* value	Adjusted odds ratio	*P* value
Gender (female vs. male)	1.050 (0.793–1.389)	*P* = 0.735		
Age (per year increase)	1.001 (0.991–1.010)	*P* = 0.900		
Creatinine (per µmol/L increase)	1.001 (1.000–1.002)	*P* = 0.024	1.001 (1.000–1.002)	*P* = 0.029
Respiratory disease (yes vs no)	0.997 (0.706–1.409)	*P* = 0.988		
Dialysis (Yes vs No)	1.404 (0.627–3.143)	*P* = 0.408		
Diabetes ( NYHA )	0.569 (0.427–0.758)	*P* < 0.001	0.536 (0.397–0.724)	*P* < 0.001
Smoking ( NYHA )	0.756 (0.584–0.979)	*P* = 0.034	0.783 (0.600–1.024)	*P* = 0.074
BMI (per kg/m^2^ increase)	0.978 (0.976–1.000)	*P* = 0.055		
TBSA (per m^2^ increase)	0.938 (0.531–1.658)	*P* = 0.827		
NYHA				
Class 1	1.279 (0.994–1.646)	*P* = 0.056		
Class 2	0.853 (0.645–1.128)	*P* = 0.265		
Class 3	0.880 (0.639–1.212)	*P* = 0.434		
Class 4	0.851 (0.473–1.532)	*P* = 0.591		
Preoperative Hb (per g/L increase)	0.991 (0.984–0.998)	*P* = 0.014	0.995 (0.987–1.002)	*P* = 0.172
Antiplatelet use < 24 h ( yes vs. no )	0.977 (0.679– 1.408)	*P* = 0.902		
Anticoagulant use < 24 h ( yes vs. no )	1.327 (0.995–1.770)	*P* = 0.054		
Non valve/CABG ( yes vs. no )	1.641 (1.210–2.225)	*P* < 0.001	1.418 (0.991–2.203)	*P* = 0.056
Previous cardiothoracic intervention ( yes vs. no )	2.412 (1.800–3.230)	*P* < 0.001	2.370 (1.748–3.215)	*P* < 0.001
Presence of cardiogenic shock	4.686 (2.706–8.117)	*P* < 0.001	3.473 (1.970–6.135)	*P* < 0.001

Many factors failed to demonstrate significant association with NRBC transfusion. These were gender, age, respiratory disease, haemodialysis preoperatively, smoking, BMI, TBSA, NYHA class, antiplatelet use within 24 h of surgery, and anticoagulant use within 24 h of surgery. Interestingly, preoperative Hb, gender, age and TBSA were highly associated with RBC transfusion, however, did not demonstrate a significant association with NRBC transfusion.

### Subgroup analysis

A total of 121 patients required transfusion of 4 or more PRBC. Factors associated with large volume transfusion on multivariate analysis include raised preoperative creatinine, presence of respiratory disease, preoperative dialysis, low BMI, low preoperative haemoglobin, previous cardiothoracic intervention and the presence of cardiogenic shock preoperatively. Of note, people with preoperative cardiogenic shock were 6 times as likely and patients with preoperative dialysis were 30 times as likely to require large volume transfusion respectively (Table 5).

**Table 5:** Subgroup analysis assessing large volume transfusion (> 4 units PRBC)

Factor	Odds ratio (95% CI)	*P* value	Adjusted odds ratio	*P* value
Gender (female vs. male)	0.993 (0655–1.506)	*P* = 0.974		
Age (per year increase)				
Creatinine (per µmol/L increase)	1.003 (1.002–1.004)	*P* = 0.001	1.007 (1.004–1.010)	*P* < 0.001
Respiratory disease ( yes vs. no )	2.049 (1.335–3.146)	*P* = 0.001	1.872 (1.170–3.000)	*P* = 0.009
Dialysis (yes vs. no )	4.103 (1.809–9.306)	*P* < 0.001	30.24 (2.549–358.7)	*P* = 0.007
Smoking (yes vs. no)	0.937 (0.637–1.378)	*P* = 0.741		
BMI (per kg/m^2^ increase)	0.956 (0.922–0.991)	*P* = 0.019	0.950 (0.914–0.987)	*P* = 0.008
TBSA (per m^2^ increase)	0.706 (0.304–1.637)	*P* = 0.417		
NYHA class 4	1.194 (0.780–1.829)	*P* = 0.872		
Preoperative Hb (per g/L increase)	0.967 (0.957–0.976)	*P* < 0.001	0.971 (0.961–0.982)	*P* < 0.001
Antiplatelet use < 24 h ( yes vs. no )	1.063 (0.504–2.246)	*P* = 0.872		
Anticoagulant use < 24 h ( yes vs. no )	1.194 (0.780–1.829)	*P* = 0.414		
Non valve/CABG ( yes vs. no )	1.259 (0.754–2.102)	*P* = 0.378		
Previous cardiothoracic intervention ( yes vs. no )	2.877 (1.934–4.281)	*P* < 0.001	2.578 (1.681–3.953)	*P* < 0.001
Presence of cardiogenic shock preoperatively (yes vs. no)	7.260 (4.480–11.77)	*P* < 0.001	6.329 (3.788–10.53)	*P* < 0.001

## Discussion

Cardiac surgery is associated with a high risk of perioperative blood loss and allogeneic blood transfusions. This is multifactorial, involving the patient’s exposure to cardiopulmonary bypass, anticoagulation, invasiveness of the procedures and coexisting comorbidities of the patients undergoing cardiac surgery. At the same time, blood transfusion in cardiac surgery is associated with adverse outcomes [19–21]. Identifying at risk patients in cardiac surgery is beneficial as prophylactic actions can be taken in a preoperative setting to minimize their need for transfusion. This study looks exclusively at preoperative factors associated with both blood loss and coagulopathy. In our study, 28% of patients required at least one unit or RBC and 18% required clotting products. This is consistent with other studies, both Australasian and international [1, 16, 22, 23]. These same studies also demonstrate significant variability in transfusion rates across centres [12, 16, 22, 23].

Patient age was a predictor of RBC transfusion on multivariate analysis. This result is also consistent with the current literature [1, 13, 15–17]. Niv et al. examined age as a predictor of blood transfusion in a retrospective cohort study. They demonstrated that age was a robust predictor of transfusion, as patients over the age of 75 had an odds ratio of 1.39 compared to those under 75 for requiring transfusion [15]. A multitude of other factors are implicated in this, including lower BMI and TBSA, higher prevalence of renal impairment and congestive cardiac failure present in the elderly population undergoing cardiac surgery [15]. Our study produced an adjusted odds ratio of 1.018 equating to a 1.8% increased risk of requiring RBC for every year increase in age. This suggests that age may be an independent predictor of transfusion risk despite the complex interplay of other factors. One potential explanation for the above is more liberal transfusion targets in the elderly to facilitate rehabilitation producing higher rates of RBC transfusion [15]. Patient age had no significant association with NRBC transfusion.

Gender is also a significant factor affecting transfusion, with female gender associated with an increased risk of RBC transfusion on multivariate analysis. This result is consistent with previous studies that demonstrate female gender as a predisposing factor for transfusion with varying levels of significance [12, 13, 16, 17, 24]. The link between gender and transfusion risk goes beyond that of cardiac surgery [25]. One theory is that a lower overall TBSA and BMI in females results in a more profound effect caused by haemodilution on cardio-pulmonary bypass (CPB) [16, 17]. Despite accounting for the effects of TBSA and BMI, female gender still is a significant risk factor for RBC transfusion. The same association cannot be found with NRBC transfusion in our study.

Evidence exists linking renal dysfunction and poor outcomes in cardiac surgery [26–28]. As such, creatinine clearance is incorporated in to preoperative risk models such as the Society of Thoracic Surgeons (STS) score and EuroSCORE [26, 27]. Higher preoperative creatinine clearance is also associated with a higher risk of requiring allogeneic blood products. Parr et al. demonstrated that a creatinine clearance of greater than 150 g/dL put patients at three times the risk of requiring RBC transfusion compared to those with a creatinine clearance of less than 100 g/dL [13]. Likewise, a high creatinine clearance was associated with a risk of requiring NRBC products on univariate but not multivariate analysis in this study [13]. Moskowitz et al., in a prospective cohort study assessed risk factors associated with bleeding, demonstrating that 28% of patients with creatinine greater than 130 µmol/L were transfused compared with only 5% of patients with creatinine of less than 130 µmol/L [12]. Our study adds to the growing body of evidence that a higher preoperative creatinine clearance is associated with an increased risk of requiring allogeneic blood products. Similarly, preoperative creatinine clearance is associated with a greater risk of NRBC transfusion on multivariate analysis. Unique to our study was the assessment of patients requiring haemodialysis preoperatively. This cohort demonstrated the strongest predictor of requiring a RBC transfusion, with an adjusted odds ratio of 5.64. Postulated mechanisms of perioperative bleeding include platelet dysfunction, impaired platelet and endothelial interactions in patients with acute renal failure [29]. Also, renal dysfunction results in erythropoietin deficiency and preoperative anaemia. As a result, reno-protective measures preoperatively are paramount in cardiac surgery, such as the cessation of nephrotoxic medications and delaying elective procedures in patients with an acute kidney injury.

Decreased TBSA and BMI demonstrated a significant risk factor for RBC transfusion in univariate analysis, however, only TBSA reached significance on multivariate analysis. We demonstrated that every unit decrease in TBSA (m^2^) there was a 2.8 times higher risk of requiring RBC. Parr et al. demonstrated a similar result on univariate analysis, however the effect was not as profound on multivariate analysis with an odds ratio of 0.58 (95% CI 0.36–0.94) [13]. This result is not surprising. McDonald et al. demonstrated that the risk for intra-operative transfusion was predicted by a combination of a patient’s preoperative haemoglobin and their TBSA in an entity termed the Transfusion Predictor Product (TPP) [4]. Patients with a smaller TBSA have a lower circulating blood volume, and therefore are prone to haemodilution on CPB [4]. Though other studies demonstrated BMI to be a significant predictor of RBC transfusion, our study failed to demonstrate that BMI is significantly associated with RBC transfusion on multivariate analysis [30]. This may mean that TBSA rather than BMI is a more accurate predictor of circulating blood volume and therefore predict the haemodilutary effects of CPB. Our study demonstrated that neither TBSA nor BMI significantly predicted the use of NRBC use in cardiac surgery. Consideration should be given to reducing the haemodilution effects in these patients with the use of small volume circuits or retrograde priming of CPB circuits.

Preoperative anaemia has been strongly associated with RBC transfusion in cardiac surgery [31]. Some preoperative risk scores incorporate Hb level into a risk equation predicting risk of RBC transfusion in cardiac surgery [4, 12]. Our study demonstrated that for every unit increase in Hb there was a 2% decrease in the risk of necessitating RBC transfusion. This result was highly significant on both univariate and multivariate analysis. Huang et al. have published similar significant results in a retrospective cohort study, whereby the risk of massive transfusion increased by 50% with a 10 g/L decrease in haemoglobin [30]. Therefore the optimisation of preoperative Hb is crucial. The European Association of Cardiothoracic Surgery (EACTS) guidelines published Grade 2 evidence for the use of erythropoietin or iron transfusion preoperatively to optimise haemoglobin levels [32]. An increase in Hb is also associated with a decrease in the risk of requiring NRBC products, however this only reached significance on multivariate analysis.

Medications that affect the coagulation system have been show to increase bleeding and transfusion rates in some studies [1, 33]. Others, however, have not demonstrated a significant impact on the risk of transfusion [12, 13, 30]. Our study demonstrates that the use of Ticagrelor or Clopidogrel within 24 h of operating is not significantly associated with an increased risk of either RBC or NRBC transfusion. Other retrospective cohort studies have also demonstrated that exposure to antiplatelet medications does not have a significant impact on the rate of either RBC or RBC transfusions [12, 13]. That being said, a recent randomised control trial conducted by Gherli et al. [33] demonstrated that the use of preoperative Ticagrelor with aspirin, compared to aspirin alone is associated with a greater risk of platelet transfusion. In addition, patients who received Ticagrelor up to the time of surgery were at increased risk of severe bleeding [33]. As a result, a Level B recommendation exists for the cessation of Ticagrelor at least 3 days, and Clopidogrel at least 5 days prior to elective cardiac surgery. Our study does show that the use of agents within 24 h cardiac surgery significantly increases the risk of RBC transfusion, with an odds ratio of 1.302 on multivariate analysis. Anticoagulant use was also significantly associated with an increased risk of NRBC products on univariate analysis but this failed to reach significance on multivariate analysis.

The presence of cardiogenic shock preoperatively was significantly associated with an increased risk of RBC transfusion, with an OR of 3.257 on multivariate analysis. It also significantly predicts the risk of NRBC transfusion with an OR of 3.473. Time constraints and the inability to correct pre-existing anaemia, or renal injury in this cohort of patients may render them prone to necessitating blood products [12]. Resuscitation of patients who suffered an acute cardiac event may result in haemodilution and therefore increase the risk of RBC and NRBC transfusion. Alternatively, the threshold to transfuse shocked patients may be lower. Though our study is the first to explore the rate of transfusion associated with preoperative cardiogenic shock, others have demonstrated a significant increase in the rate of transfusion associated with emergent procedures [12, 13]. The presence of a significant finding on multivariate analysis indicates that cardiogenic shock in the preoperative setting is an independent risk factor for allogeneic blood transfusion.

Patients who have had previous cardiothoracic interventions also demonstrated a significant risk of requiring RBC transfusion, with an OR of 1.563 on multivariate analysis. Patients with a history of previous cardiothoracic interventions have an even greater risk of requiring NRBC products with an OR of 2.370. This finding is consistent with a number of other retrospective studies [12, 13, 21, 30]. Reoperations are associated with an increased risk of bleeding and coagulopathy [12, 21]. Reoperations are also more technically challenging, exposing patients to greater CPB times [12]. In patients undergoing redo surgery it is doubly important to address reversible factors prior to surgery to minimise the risk of requiring transfusion. Likewise, patients who have not undergone a valvular or bypass operation demonstrated an OR of 1.567 for requiring RBC post procedure on multivariate analysis. The type of cardiac procedure has been a factor contributing to bleeding, and has been explored in preoperative risk prediction models [34, 35]. Karkouti et al. demonstrated that patients that underwent combined Valve/CABG procedures had a significantly increased risk of requiring a massive blood transfusion (i.e., greater than five units of RBC) in a prospective cohort study of 10,667 patients. In this study, procedures other than isolated CABG/single Valve procedure were associated with an OR of 1.36 (95% CI 1.04–1.79, *P* = 0.03) [36]. This result is not surprising. Complex aortic procedures, urgent presentations, prolonged CBP use and hypothermic arrest all account for a higher risk and therefore a greater likelihood of exposure to blood products [36].

Interestingly a past or present history of smoking and current history of diabetes both appeared to be a protective factor towards NRBC transfusion on univariate analysis. The history of Diabetes Mellitus was a significantly associated with a decreased risk of NRBC transfusion on multivariate analysis. This finding has been alluded to in previous studies [17, 37]. Smoking gives rise to polycythaemia and also induces a hypercoagulable state, which may reduce the risk of bleeding [38]. Diabetes can induce a prothrombotic state also may reduce the risk of bleeding. Our study did not assess mortality and morbidity however, and it is important to note that diabetes and smoking are associated with overall increased rates of complications post operatively.

In our study, a total of 121 patients required large volume transfusion (i.e., defined as four or more units of PRBC), or 7.5% of patients. In other studies, approximately 21% of the cohort required massive transfusion [30]. Factors associated with large volume transfusion reported in literature include low BMI, advanced age, renal dysfunction, low haemoglobin and female gender [30]. Our study was novel in its reporting of the association of large volume transfusion with the presence of preoperative cardiogenic shock and redo surgery.

Our study demonstrated a number of significant preoperative factors associated with both RBC and NRBC product transfusion in cardiac surgery. Limiting analysis to preoperative factors that are easily assessable in a preadmission setting allows us to risk stratify patients before an operation. However these results must be interpreted in light of the limitations of the study. The retrospective cohort nature of this study does not allow us to control for selection bias. Also the factors that were assessed were only done so within the confines of the ANZSCTS database and its definitions. As an example, the database does not record preoperative haematocrit, coagulation profile or platelet count; factors that are significantly associated with bleeding in other studies [12, 13, 16, 17, 30]. Assessing these factors would prove useful and could be achieved with a prospective study design. Likewise, the results obtained from the ANSZCTS database can be subject to data entry error despite attempts made to externally validate the database. Our study assessed cardiac surgery as a whole, without the exclusion of emergent procedures, aortic procedures or patients with infective endocarditis. Other studies accounted for CPB time, use of CPB and emergent procedures [14, 16, 17, 30]. These are admittedly important factors to assess as aortic operations and valvular operations associated with endocarditis are associated with a greater bleeding risk [12, 13]. That being said, these only accounted for 6.7% of our patient cohort in this study, with the significant majority of operations being CABG, valve or a combined CABG and valve procedure.

## Conclusion

These findings from a single Western Australian institution demonstrated a number of significant preoperative factors associated with both RBC and NRBC transfusion in patients undergoing cardiac surgery. Patient age, preoperative creatinine clearance, dialysis, TBSA, preoperative, Hb, history of previous cardiac interventions and the presence of cardiogenic shock preoperatively were all significantly associated with RBC transfusion on multivariate analysis. Patients on dialysis and the presence of cardiogenic shock preoperatively both demonstrated the greatest risk, with adjusted OR of 5.643 and 3.257 respectively. Preoperative creatinine clearance, dialysis and the presence of cardiogenic shock were all significantly associated with NRBC use on multivariate analysis. Patients who have had a previous cardiothoracic intervention are associated with a greater risk of RBC and NRBC transfusion, with adjusted OR of 1.563 and 2.370 respectively. Large volume transfusion occurred in 7.5% of patients. These results provide useful insights for identifying and managing patients at higher risk of requiring blood products in cardiac surgery prior to their operation.

## Data Availability

The datasets used and/or analyzed during the current study are available from the corresponding author on reasonable request.

## Abbreviations

RBC: Red blood cell
NRBC: Non red blood cell
FFP: Fresh frozen plasma
ANZSCTS: Australia and New Zealand Cardiac Surgery Database
OR: Odds ratio
BMI: Body Mass Index
TBSA: Total body surface area
NYHA: New York Heart Association
CABG: Coronary artery bypass graft
Hb: Haemoglobin
CPB: Cardiopulmonary bypass
STS: Society of thoracic surgeons
TPP: Transfusion predictor product
EACTS: European Association of Cardiothoracic Surgery
